# The influence of systematic heat and drought applications at defined growth stages on malting barley starch properties

**DOI:** 10.1002/jsfa.14183

**Published:** 2025-04-02

**Authors:** Veronika Franz, Stefan Hör, Hannes Petermeier, Christoph Neugrodda, Thomas Becker, Martina Gastl

**Affiliations:** ^1^ Chair of Brewing and Beverage Technology Technical University of Munich Freising Germany; ^2^ Research Center Weihenstephan for Brewing and Food Quality Technical University of Munich Freising Germany; ^3^ Associate Professorship of Biostatistics (Prof. Ankerst) Technical University of Munich Freising Germany

**Keywords:** differential scanning calorimetry, drought stress, gelatinization, heat stress, malting barley, starch

## Abstract

**BACKGROUND:**

Starch gelatinization behavior and the proportion of B‐granules are crucial for malt quality as a result of their impact on starch degradation during malt‐based beverage production. Starch structure and resulting starch properties are determined by the starch synthesis during barley plant growth, which is influenced by barley genetics and environmental growing conditions. Because climate change is altering growing conditions increasingly, insights into the relationship between growth conditions, genetics and relevant starch properties are of upcoming importance. Thus, as a factorial experiment, three malting barley varieties (Morex, Scarlett and Avalon) were grown in highly controlled climate chambers with systematic variations in heat and drought conditions to investigate the influence of (i) pre‐anthesis growth temperature [growth stage (GS) < 61]; (ii) temperature from GS 61–75 and from GS 75–93; (iii) malting barley variety; (iv) drought stress; and (v) interactions between those factors on resulting gelatinization behavior (using differential scanning calorimetry) and the B‐granule proportion (detected by laser diffraction).

**RESULTS:**

(Stepwise) multiple linear regression calculations (MLR/SMLR) revealed that (i) pre‐anthesis growth temperature significantly affected gelatinization behavior (onset, *T*_o_; endset, *T*_E_; enthalpy, Δ*H*) but not B‐granule proportion; (ii) starch properties were not significantly and variety‐independently different after heat stress from GS 61–75 or heat stress from GS 75–93; and (iii) additional drought stress did not significantly change *T*_o_ and *T*_E_ values *per se*, but significantly affected Δ*H* and B‐granule proportion values.

**CONCLUSION:**

Growth conditions before GS 55 significantly impact starch gelatinization behavior, indicating that starch synthesis is highly affected by conditions before anthesis. © 2025 The Author(s). *Journal of the Science of Food and Agriculture* published by John Wiley & Sons Ltd on behalf of Society of Chemical Industry.

## INTRODUCTION

Starch is an essential component of brewing malt, with barley (*Hordeum vulgare* L.) being the most common grain for brewing worldwide. The breakdown of starch into fermentable sugars during mashing, called saccharification, is mandatory for further processing (i.e. high extract yields) and the quality of the finished beer.^1^, ^2^

Starch consists of the two polysaccharides, amylose and amylopectin, which are both poly‐glucans. In linear amylose, the glucose monomers are connected only by α‐1,4‐glycosidic bonds, whereas amylopectin is branched by additional α‐1,6‐glycosidic bonds. On the macroscopic level, barley starch is stored in A‐ and B‐granules. A‐granules have a diameter of 10–30 μm and on average account for 85–90% of the total starch weight. B‐granules have a diameter of up to 6 μm.^3^ Although the B‐granule ratio in barley starch is typically 10–15%, it can vary between 5% and 35% depending on the growth conditions.^4^, ^5^ The physicochemical properties of starch, most notably the gelatinization behavior, have a crucial impact on saccharification during the mashing process. Gelatinization, the melting of crystalline structures within the starch granules, is necessary for the rapid and complete degradation of starch by the cereal‐intrinsic amylolytic enzymes.^6^ The gelatinization behavior therefore is a crucial quality parameter for malting barley.^7^, ^8^ Using differential scanning calorimetry (DSC) allows the characterization of the gelatinization process of purified starch in great detail by measuring the energy needed to heat both the starch sample and a control at the same rate. It determines the beginning of gelatinization as onset‐temperature (*T*_o_) and the end of gelatinization as endset‐temperature (*T*_E_). Furthermore, the enthalpy (Δ*H*) can be determined, making it an impactful analysis.^9^, ^10^ Also, the macroscopic structure of starch, primarily the B‐granule proportion, may impact the saccharification behavior during brewing as B‐granules can show up to 5 °C higher gelatinization temperatures compared to A‐granules. Especially in high‐gravity mashes, a high B‐granule proportion can be unfavorable for the conversion of starch to fermentable sugars.^11^, ^12^, ^13^, ^14^

The structure of starch, determining the gelatinization behavior of the mature grain,^14^, ^15^ is developed during starch synthesis, taking place when kernels are formed during barley plant growth. Although plant development happens at a different pace in every barley variety, the Zadok growth stages (GS) can ensure comparability of different varieties by evaluating the plants at the same growth stage rather than the same growing time. The GS characterize plant development,^16^ dividing the process into 10 sections. Namely, these are germination (00–09), seedling growth (11–19), tillering (21–29), stem elongation (31–39), booting (41–49), inflorescence emergence (51–59), anthesis (61–69), milk development (71–79), dough development (81–89) and ripening (91–99). Each section is divided again to describe the plant development in more detail. In barley, starch synthesis starts shortly after the anthesis stage^17^, ^18^ and is performed in four distinct steps: elongation, branching, debranching and crystallization.^17^, ^18^, ^19^ Apart from crystallization, these steps are catalyzed by specific enzymes.^19^, ^20^, ^21^ In the elongation process, starch synthases connect glucose monomers by α‐1,4‐glycosidic bonds, building linear polymers. Amylose synthesis consists of only the elongation step. For amylopectin synthesis, a part of the glucose chain is cut off and reconnected to the chain as a branch by α‐1,6‐glycosidic bonds. Several different branching enzymes perform this process. The subsequent debranching removes all side chains that are unfavorable for crystallization.^17^, ^18^, ^19^, ^20^, ^22^ In the crystallization step, the linear sections of the α‐glucan chains form double helixes which again form the crystalline layers of the starch granules by building either A‐ or B‐type crystal conformations. A‐type crystal conformation binds less water molecules and has a higher enthalpy. B‐type crystal conformation can reorganize into A‐type crystal conformation under hot and dry conditions; however, this transition is irreversible.^23^

**Table 1:** Overview of the independent variables used for MLR (multiple linear regression) and SMLR (stepwise multiple linear regression) analyses

Variables for SMLR				
Interactions				MLR
Variety‐related			Others	Variables
AV:mm	MO:mm	SC:mm	RN:mm	AV
AV:mh	MO:mh	SC:mh	RN:mh	SC
AV:hm	MO:hm	SC:hm	RN:hm	MO
AV:hh	MO:hh	SC:hh	RN:hh	
AV:RN:mm	MO:RN:mm	SC:RN:mm	IR:mm	mm
AV:RN:mh	MO:RN:hm	SC:RN:mh	IR:mh	mh
AV:RN:hm	MO:RN:mh	SC:RN:hm	IR:hm	hm
AV:RN:hh	MO:RN:hh	SC:RN:hh	IR:hh	hh
AV:IR	MO:IR	SC:IR	RN:hm:IR	IR
AV:RN:IR	MO:RN:IR	SC:RN:IR	RN:mh:IR	RN
AV:RN	MO:RN	SC:RN	RN:mm:IR	
			RN:hh:IR	
			RN:IR	

Abbreviations: AV, Avalon; SC, Scarlett; MO, Morex; RN, run; IR, irrigation; mm, moderate–moderate; mh, moderate–hot; hm, hot–moderate; hh, hot–hot.

The expression and activity levels of all involved enzymes determine the final starch structure. Climate conditions trigger regulatory answers, altering those levels and resulting in a different starch structure.^5^, ^24^, ^25^, ^26^, ^27^ Most studies observe the impact of stress in the time of GS 61 (begin of anthesis) to 99 (fully matured grain)^5^, ^24^, ^27^, ^28^, ^29^, ^30^ because, during those stages, the enzymes involved in starch synthesis are most active.^31^, ^32^, ^33^ However, there is evidence that temperature stress before anthesis impacts grain development^34^, ^35^, ^36^ and hence starch synthesis might also be affected.

Heat or drought conditions occur more often in recent years as a result of climate change. These have been shown to impact starch synthesis and result in starch structures that gelatinize at higher temperatures in several crops such as wheat, rice, potatoes and maize^37^, ^38^, ^39^, ^40^, ^41^, ^42^, ^43^, ^44^ and have proven to be unfavorable for the gelatinization behavior in malting barley.^45^ The starch in heat and drought‐stressed crops also shows an altered B‐granule proportion,^28^, ^46^ lower amylose content^41^, ^47^ and a higher ratio of long amylopectin side chains.^27^, ^30^, ^44^, ^48^, ^49^ Drought, which is often applied in addition to heat stress, does impact grain yield; however, there is evidence that drought stress alone does not affect starch gelatinization behavior.^4^, ^38^ It has been shown that heat stress at the beginning of the grain‐filling phase has a more significant impact on grain filling than the comparable heat stress at a later point.^50^ However, because different crops and even different varieties of the same crop show significantly different stress responses,^44^ a simple knowledge transfer from other crops or non‐malting barley varieties will not suffice.

Thus, the present study tests the following hypotheses: (i) starch properties are affected only by growing temperatures during grain filling (GS 61–99); (ii) heat stress at the beginning of grain filling (GS 61–75) impacts starch properties more than heat stress at the end of grain filling (GS 75–92); and (iii) the combination of drought stress with heat stress affects starch properties in malting barley. For that, three different malting barley varieties were cultivated under defined heat and drought stress conditions in climate chambers, and the resulting starch was characterized in terms of gelatinization behavior and B‐granule proportion. Multiple linear regression methods were used to identify significant dependencies between starch properties, varieties and growing conditions based on a factorial experimental design.

## MATERIALS AND METHODS

### Selection of malting barley varieties

To guarantee varieties with high malting and brewing quality, as well as a high genetic difference, the following malting barley varieties were selected: Morex (MO), Scarlett (SC), and Avalon (AV). MO was released in 1978 as a six‐row malting barley variety and was successful in North America, especially because of its high extract yields in brewing. SC was the variety with the largest worldwide acreage in the 1990s and is, at this time, notable for its low protein content. AV was released in 2012 in Germany and was one of the leading varieties in Europe between 2015 and 2020. It is special for its balanced cytolytic and proteolytic activity during malting. These varieties were selected to ensure both purebred strains explicitly tailored for the malting and brewing industry and to achieve a high genetic diversity through intermittent breeding successes.

### The factorial design of the growing experiments

The study of how climate scenarios affect the introduced starch properties was conducted using a factorial design, with variety (VR), pre‐anthesis growing temperature [run (RN)], temperature scenario from GS 61–93 (TS) and irrigation between GS 61–93 (IR) seen as independent factors, as detailed below.

#### General procedure

To grow barley plants under defined growth conditions during grain‐filling, they were raised in greenhouses (Greenhouse Laboratory Center Dürnast, TUM, Weihenstephan, Germany) under daily irrigation until reaching GS 55. Subsequently, the plants were transferred to climate chambers (TUMMesa, Weihenstephan, Germany), where they continued to grow until maturity under precisely controlled environmental conditions. Both, the growth temperature and irrigation were varied as defined. The experiment was conducted twice (RN 1 and 2) with different growth temperatures up to GS 55 in the greenhouses as a result of seasonal variation, whereas the conditions in the climate chambers remained the same for both runs.

#### Raising and greenhouse conditions

Barley kernels were germinated in the laboratory for 2 days before seeding. Therefore, 300 g of z‐seeds were steeped for 5 h in 500 mL of H_2_O (20 °C) in a 2‐L laboratory beaker. Water was poured off, and the wet kernels were stored for 19 h in the same beaker at room temperature. The same procedure was repeated the next day. To guarantee the development of the same amount of main shoots in every pot, only kernels with a visible sprout were sown into the soil (seven seeds per 3‐L pot, filled with standardized peat soil substrate C700 (Stender GmbH, Schermbeck, Germany)). 64 pots represented each variety.

**Figure 1:**
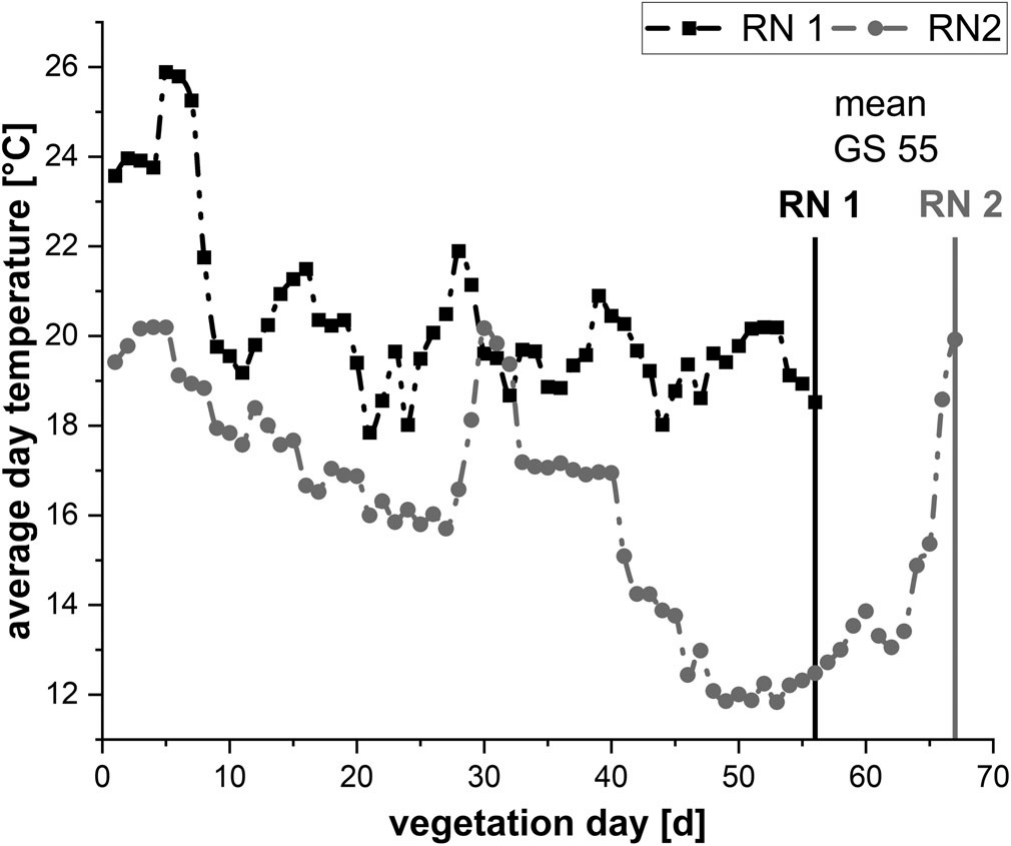
Average day temperatures in the greenhouse from sowing until mean growth stage (GS) 55. RN 1 (56 vegetation days) experienced higher temperatures (average pre‐anthesis day temperature: 20.37 °C), so plant development was significantly quicker than RN 2 (67 vegetation days; average pre‐anthesis day temperature: 15.99 °C). RN, run. GS, growth stage.

The plants, then, were grown on flooding tables being watered once a day for 1 h by defined flooding. To control soil nutrients and keep the nitrogen content in mature barley grains below 11%, as recommended for malting barley,^51^ fertilization was adjusted according to a previous study.^7^ RN 1 was sown on 8th September 2022 and RN 2 on 26th October 2022 to achieve different temperature conditions until GS 55 (Fig. 1). The growth stages of the developing plants were documented weekly. As soon as more than half of all plants reached GS 55 (RN 1: 56 days after sowing; RN 2: 67 days after sowing), all the pots were transferred to TUMMesa climate chambers.

#### Conditions in the climate chambers

**Figure 2:**
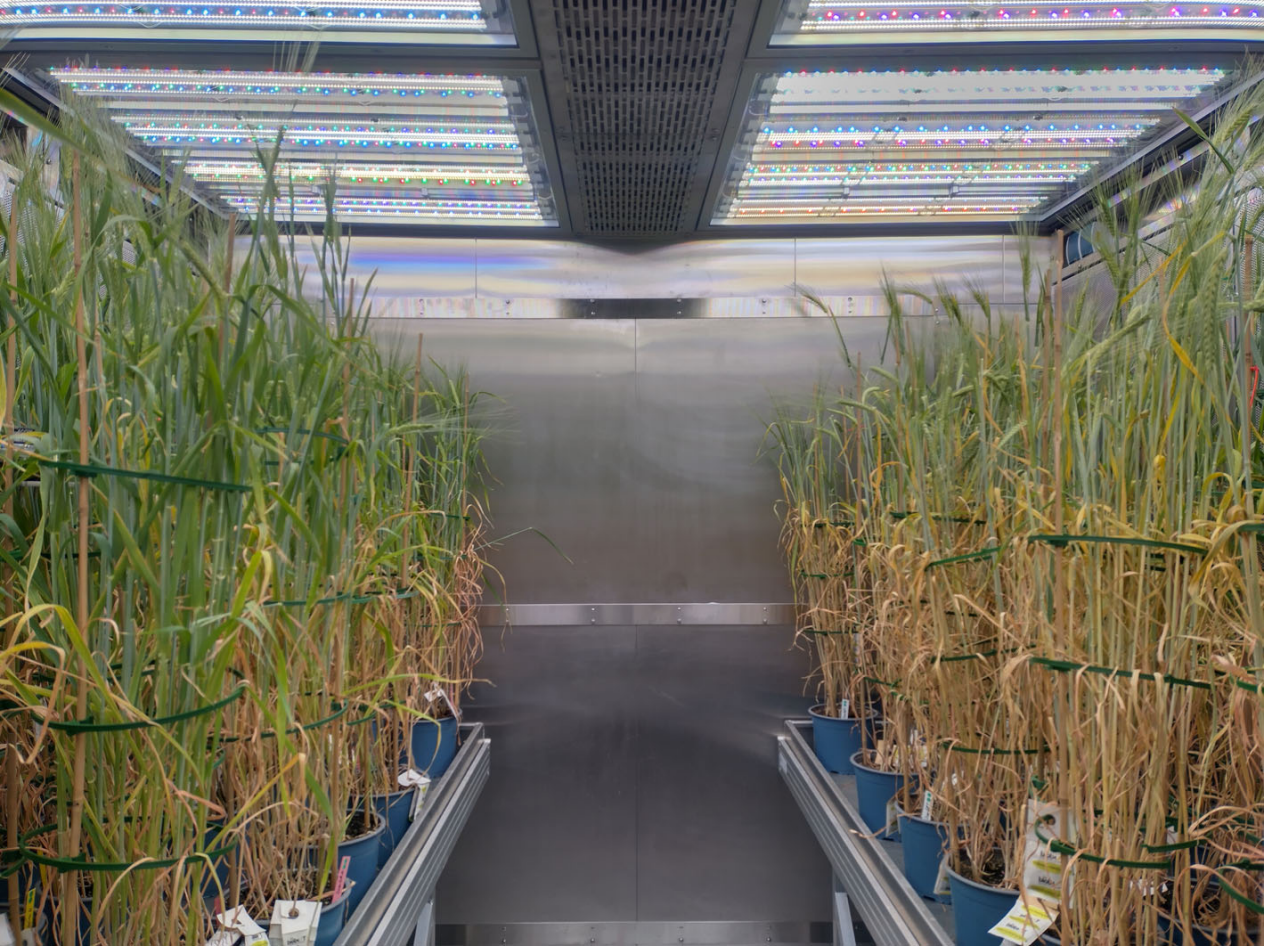
Barley plants at on average growth stage 80 in TUMMesa climate chambers; on the left irrigated plants; on the right drought‐stressed plants.

The climate chambers at TUMMesa (Fig. 2) are engineered by regineering GmbH (Pollenfeld, Germany) and allow the precise control of temperature, relative humidity, light, and CO_2_. The LED system (Vossloh Schwabe, Urbach, Germany) comprises 10 individually controllable sub‐systems for sunlight simulation. Several recently published studies verify the accuracy and broad applicability of the measurement and control technology of these climate chambers.^52^, ^53^, ^54^, ^55^, ^56^, ^57^

The climate chambers used for this study were parametrized according to the following conditions: 450 ppm CO_2_, 9 h night (0 PAR), 13 h daylight (325 PAR), 1 h sunset (constant linear adjustment from day to night conditions), 1 h sunrise (constant linear adjustment from night to day conditions). Relative humidity was adjusted to the different temperature treatments to ensure an equal transpiration rate. The chambers for moderate growth temperature were set to 17/22 °C respectively 86/40% for 9/13 h, the chambers for hot growth temperature were set to 25/28 °C respectively 77/58%. Grain‐filling was divided into Phase 1 (GS 61–75) and Phase 2 (GS 75–93). By moving the plants between the hot and moderate chamber between Phase 1 and Phase 2, four temperature scenarios (TS) during grain filling were simulated: moderate–moderate (mm), moderate–hot (mh), hot–moderate (hm) and hot–hot (hh). Irrigation (IR) was adjusted by daily watering either to a soil water content of 10–25% (irrigated) or 3–5% (drought), measured by COMBI MST 5000 (STEP Systems GmbH, Nürnberg, Germany). By varying irrigation (IR) and temperature scenario (TS) (mm, mh, hm and hh) during Phase 1 or Phase 2, eight different treatments per VR and RN could be studied. With 64 sown pots per VR, eight pots represented one growing scenario.

**Figure 3:**
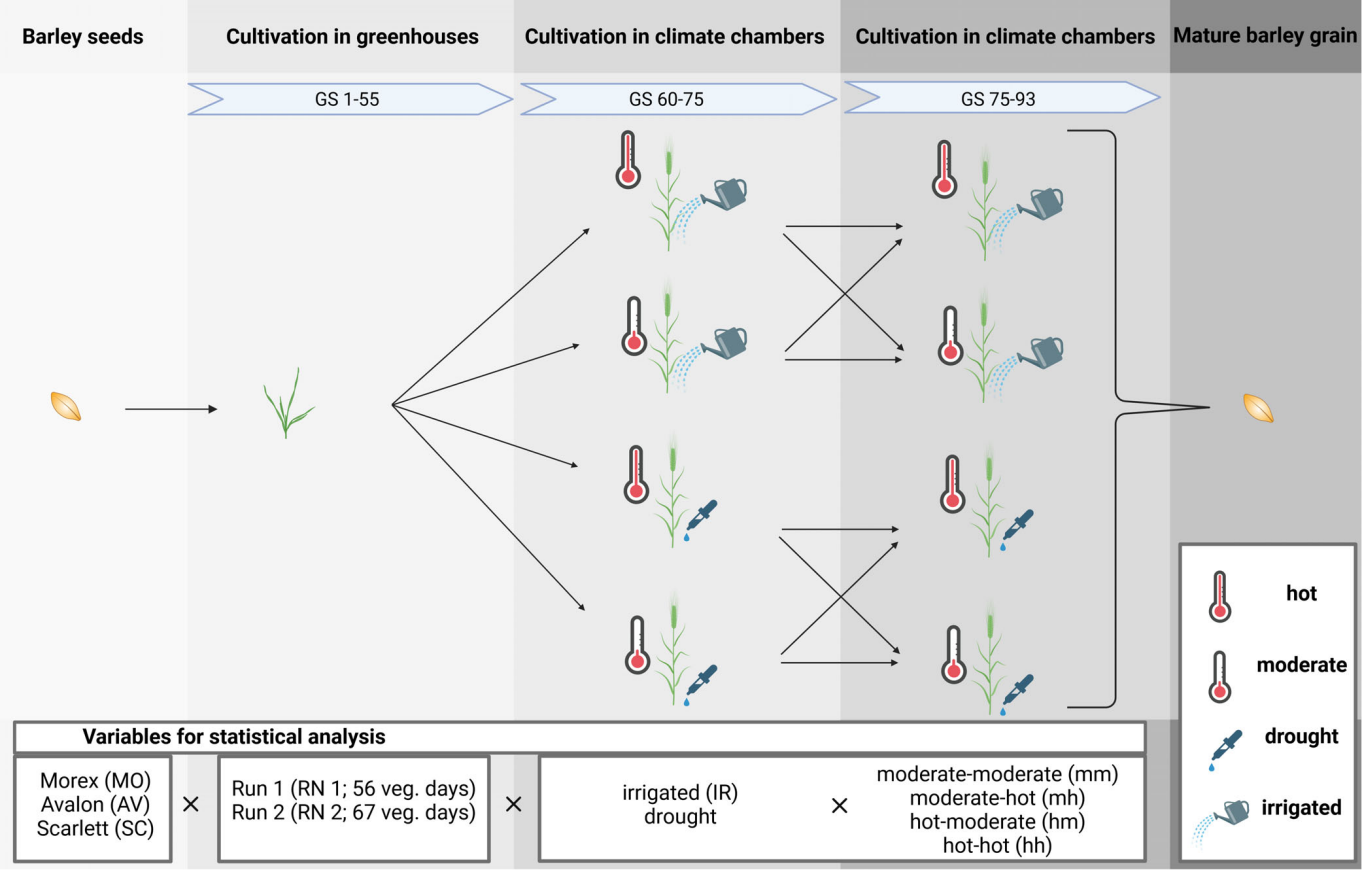
Experimental design of the growing experiments (created with BioRender.com) with variables and corresponding abbreviations for statistical analysis.

The barley grains were harvested by cutting the ears with pruning shears and threshing them using a grain threshing machine (Labordrescher LD350; Wintersteiger AG, Ried im Innkreis, Austria). To ensure sufficient material for subsequent laboratory analyses, grains from the same VR and growth conditions were pooled because some conditions, particularly the hh temperature scenario combined with drought stress, resulted in low grain yields. The pooled samples were carefully homogenized and divided into subsamples for analysis. The experimental design is illustrated in Fig. 3.

### Analytics

#### Starch purification

For starch extraction the method from Rittenauer *et al*.^58^ was modified as described in the following: 2 g of milled barley (Laboratory Mill 3100; Perten Instruments Inc., Springfield, IL, USA; 0.8 mm sieve size, 16 800 rpm) were mixed with 20 mL of demineralized water in a 50‐mL tube. The pH was adjusted to 8.0 with NaOH (1 m) for proteinase K treatment. Samples were incubated at 40 °C for 5 min, then treated with 50 μL of proteinase K (AppliChem GmbH, Darmstadt, Germany) for 30 min with periodic vortexing. The pre‐treated samples were filtered through an 80 μm polyester textile filter (Schwegmann Filtrationstechnik GmbH, Grafschaft, Germany). The starch suspension was collected and washed with deionized water to 50 mL. Samples were centrifuged at 3500 × *g* for 5 min, and the supernatant discarded. The pellet was resuspended in 7 mL of deionized water and layered onto an 80% w/v CsCl column (Carl Roth GmbH & Co. KG, Karlsruhe, Germany) in a new tube. After centrifugation at 4500 × *g* for 5 min without brake, the supernatant and the CsCl column were removed. Starch was resuspended in 20 mL of deionized water, centrifuged at 3500 × *g* for 5 min, and the supernatant discarded. This extraction procedure was repeated twice per barley sample.

#### Starch characterization

##### B‐granule proportion

The starch granules' size distribution was determined by laser fraction (Mastersizer 3000 with Hydro EV; Malvern Instruments GmbH, Kassel, Germany; accuracy: ± 0.6%) just after starch purification. The wet starch pellets were homogenized by filling the tubes with 7 mL of deionized water and mixing the suspension for 1 min. Sample material was pipetted into the sample beaker of the Hydro EV until the turbidity lay in the desired obscuration range (3–10%). Each sample was analyzed twice, whereas one measurement procedure included five single laser fraction determinations, and the results were averaged. To determine the proportion of B‐granules in a sample, the values related to the volume of total starch of particle size classes up to 5.92 μm were cumulated and defined as the proportion of B‐granules. B‐granules are defined as those with a size of up to 6 μm.^3^, ^5^, ^28^

##### Gelatinization behavior

Measurements with a DSC 250 (TA Instruments, New Castle, DE, USA; temperature precision: ± 0.008 °C; enthalpy precision: ± 0.08%) were carried out to determine the starch gelatinization behavior. Knowing that the starch‐to‐water ratio as well as the heating profile significantly affects the results for *T*_o_, *T*_E_ and Δ*H*, a standardized method with a starch‐to‐water ratio of 1: 2.5 and a heating profile of 10 K min^−1^ (from 20 to 90 °C) was used analogously to a previous study^7^; *T*_o_, *T*_E_ and Δ*H* were automatically calculated from the graphs obtained using TRIOS, version 5.1.1 (UB TA Instruments, Eschborn, Germany). Moisture content of measured starches and sample weight were considered in the values' calculation.

### Statistical analysis

Statistical analyses were conducted using SPSS, version 29.0 (IBM Corp., Armonk, NY, USA). Concerning the stated hypotheses, multiple linear regression (MLR) and stepwise multiple linear regression (SMLR) were employed to assess the impact of independent variables from the factorial design (RN, VR, TS and IR) on the respective dependent variables (starch characteristics: *T*_o_, *T*_E_, Δ*H* and B‐granule proportion). Residuals were checked for normality with P‐P‐Plots while no severe departures for normality could be observed. Note that MLR/SMLR calculations were intentionally used to identify significantly influencing variables rather than developing a predictive model with the benefit of avoiding post‐hoc testing in the analysis of variance. As a result of the dummy coding of the independent variables, the influence can be read directly from the regression coefficient. Hence, the numbers in brackets (see Results) represent the respective regression coefficients.

First, MLR was performed to explore the relationship between the independent variables and the dependent variables without considering the interactions between the independent variables. Following this, SMLR was conducted to systematically evaluate the inclusion of interactions and identify the most significant independent variables while controlling for non‐significant variables. Table 1 provides an overview of all variables used for MLR and SMLR. The interactions used for the SMLR starting model were selected according to possible influences on the dependent variables. To interpret the results of the MLR and SMLR, the regression coefficient of all significant independent variables was construed as a measure of the influence on the respective dependent variable.

SMLR was conducted with a selection criterion of *P* < 0.05 (as for MLR) and a removal criterion of *P* > 0.10, ensuring the inclusion of statistically significant variables at the same time as eliminating non‐significant ones.

## RESULTS

### Impact of growth conditions on onset‐temperatures (*T*_o_)

**Figure 4:**
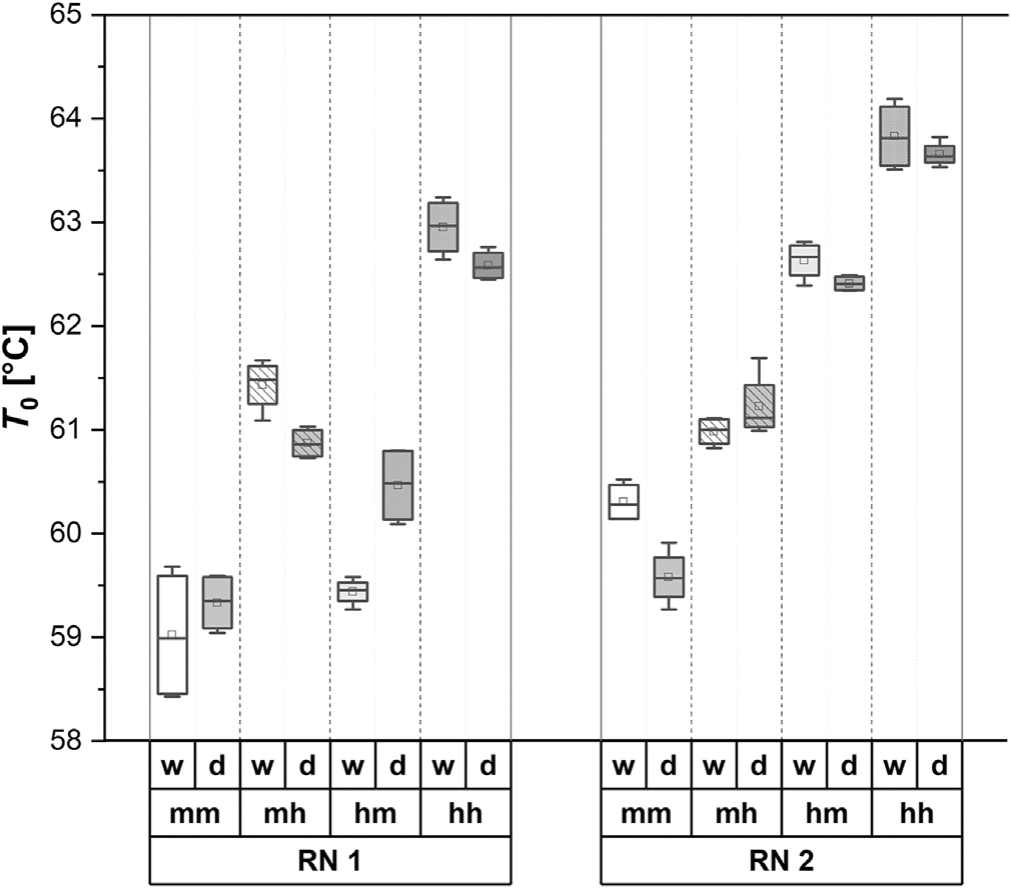
Onset‐temperature (*T*_o_) values of barley variety Avalon in dependence of the growing conditions: RN, run (expressing a difference in the pre‐anthesis growing temperature; s. Raising and greenhouse conditions); mm, moderate–moderate; mh, moderate–hot; hm, hot–moderate; hh, hot–hot; w, watered; d, drought.

Figure 4 presents the *T*_o_ values of Avalon starches depending on the growing scenarios. In the high‐temperature scenario (hh), *T*_o_ values greater than 62.5 °C are observed, regardless of irrigation (w/d) or pre‐anthesis temperature (RN). By contrast, the values in the moderate‐temperature scenario (mm) consistently are below 60.5 °C. Furthermore, significant differences as a result of drought are only observed in the scenarios RN 1–mh, RN 1–hm and RN 2–mm compared to irrigated samples. The underlying data can be found in the Supporting information (Table S1), which also includes values for all other barley varieties observed in this study.

Table 2 presents the results of the MLR and SMLR analyses conducted on the data for all varieties (see also Supporting information, Table S1), with the coefficients listed in descending order of magnitude. Within the MLR analysis (adjusted *R* ^2^ = 0.761), the variable IR (*P* = 0.986) did not significantly explain the tested *T*_o_ values, showing that irrigation *per se* does not significantly impact the resulting *T*_o_ values. The variety (MO, AV and SC), the temperature scenarios (mm, mh, hm and hh) and the temperatures until GS 55 (RN) did significantly affect the resulting *T*_o_ values, whereas the temperature scenario hh had the greatest influence (regression coefficient = 2.977).

**Table 2:** Results of MLR and SMLR analysis with *T*_o_ being the dependent variable

MLR			SMLR		
Independent variable	Regression coefficient B	SE	Independent variable	Regression coefficient B	SE
Constant term	59.135	0.160	Constant term	61.239	0.068
hh***	2.977	0.171	AV:RN:mh***	−2.217	0.240
RN***	1.801	0.121	AV:mm***	−2.062	0.142
mh***	1.145	0.171	MO:RN***	1.733	0.186
hm***	0.918	0.171	MO***	−1.684	0.158
MO***	−0.666	0.148	RN***	1.585	0.106
SC***	0.660	0.148	MO:RN:hm***	−1.583	0.220
(adjusted *R* ^2^ = 0.761)			AV:RN:mm***	−1.572	0.271
			SC:mm***	−1.322	0.111
			hh***	1.297	0.152
			MO:hh***	−1.190	0.219
			IR:hm***	−1.171	0.130
			MO:mm***	−1.144	0.176
			AV:RN:hh***	−1.109	0.265
			SC:RN:IR***	−1.034	0.142
			RN:hm:IR***	0.971	0.181
			MO:hm***	−0.967	0.173
			MO:RN:mm***	−0.919	0.230
			AV:hm***	−0.701	0.148
			SC:hh***	0.693	0.159
			RN:mm:IR**	0.507	0.151
			AV:RN*	0.499	0.221
			IR:hh***	0.466	0.111
			MO:RN:IR*	0.460	0.187
			MO:IR*	0.219	0.132
			(adjusted *R* ^2^ = 0.958)		

*Note*: DSC temperature precision: ± 0.008 °C.

Abbreviations: AV, Avalon; SC, Scarlett; MO, Morex; RN, run; IR, irrigation; mm, moderate–moderate; mh, moderate–hot; hm, hot–moderate; hh, hot–hot; MLR, multiple linear regression; SMLR, stepwise multiple linear regression; DSC, differential scanning calorimetry.

* *P* < 0.05;

** *P* < 0.01;

*** *P* < 0.001.

With an adjusted *R* ^2^ of 0.958, SMLR identified 24 variables significantly impacting the *T*_o_ values (Table 2). The comparison with the MLR results shows that the temperature scenario hh results in significantly higher *T*_o_ values regardless of the variety [SMLR (1.297) resp. MLR (2.977)]. However, the increase in *T*_o_ is significantly lower for the MO variety (MO:hh −1.190) than for AV and SC. The SMLR also shows that the other temperature scenarios mm, mh and hm identified in the MLR significantly change the *T*_o_ values, but the extent depends on the variety [e.g. AV:mm (−2.062), SC:mm (−1.322), MO:hm (−0.967), AV:hm (−0.701), SC:hh (0.693), etc.].

The temperature before GS 55 (RN) significantly influences the *T*_o_ values independent of the variety [regression coefficient SMLR (1.585)/MLR (1.801)], where the increase in *T*_o_ due to RN is higher for the MO variety than for AV or SC (MO:RN 1.733). Regardless of the temperature scenarios, MO presents significantly lower values than AV and SC (MLR: −0.666 resp. SMLR: −1.684). The generally higher *T*_o_ values for SC than AV (as shown in MLR) depend on the temperature scenarios (SC:mm −1.322; SC:hh 0.693).

With regard to the variable IR, which was classified as non‐significant in the MLR, the SMLR shows that irrigation does not have an influence on the *T*_o_ values *per se* but does have a significant effect concerning individual varieties and temperature scenarios: irrigation during the hm scenario results in significantly lower *T*_o_ values regardless of the variety (IR:hm −1.171). With a comparatively small but significant effect, irrigation in the temperature scenario hh leads to significantly higher *T*_o_ values (IR:hh 0.466).

### Impact of growth conditions on endset‐temperature (*T*_E_)

**Figure 5:**
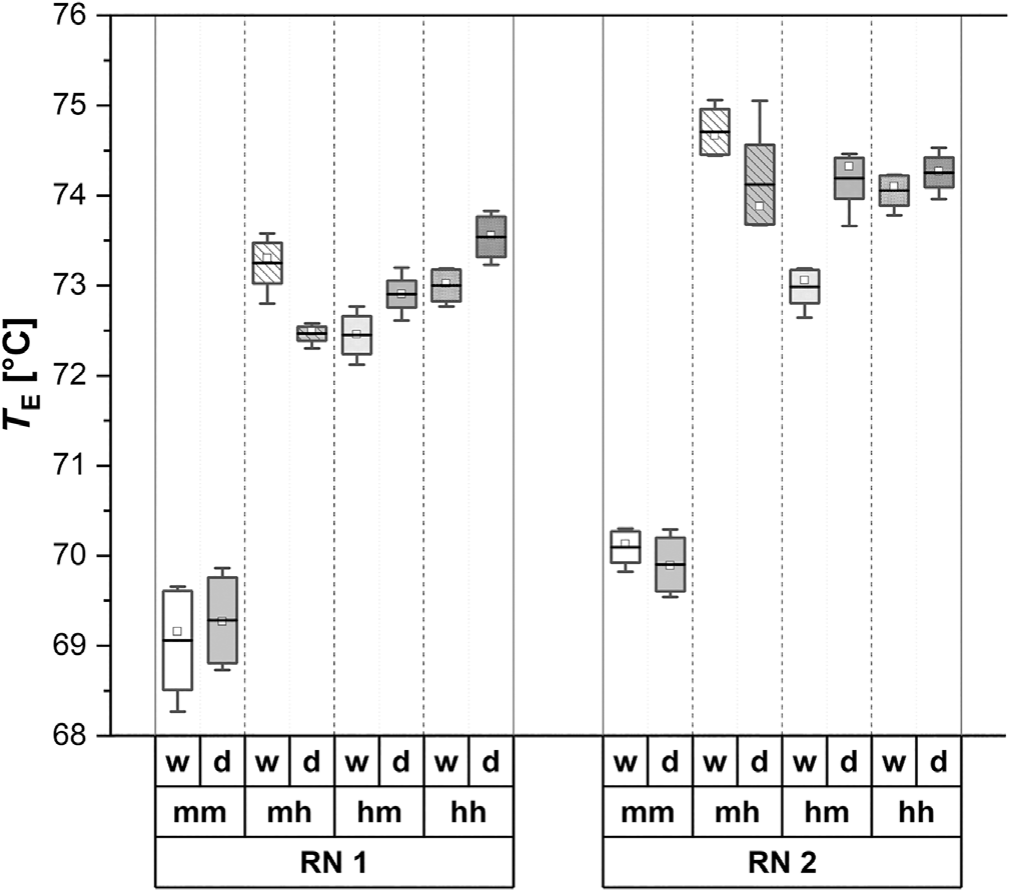
Endset‐temperature (*T*_E_) values of barley variety Avalon in dependence of the growing conditions: RN, run (expressing a difference in the pre‐anthesis growing temperature; s. Raising and greenhouse conditions); mm, moderate–moderate; mh, moderate–hot; hm, hot–moderate; hh, hot–hot; w, watered; d, drought;

Figure 5 presents the *T*_E_ values of Avalon starches, depending on the growing scenarios. In both RN 1 and RN 2, the values for the scenario mm are significantly lower (68.30–70.10 °C) compared to the mh, hm and hh scenarios (72.40–75.00 °C). Additionally, the warmer pre‐anthesis scenario (RN 1) tends to exhibit slightly lower values than the colder scenario RN 2. Similar to the *T*_o_ values, significant differences between irrigated and drought‐stressed samples are only observed in the RN 1‐mh and RN 2‐hm scenarios. The underlying data can be found in the Supporting information (Table S2), which also includes values for all other barley varieties observed in this study.

Table 3 shows the results of MLR and SMLR performed for the dependent variable *T*_E_ conducted on the data for all varieties (see also Supporting information, Table S2). All independent variables significantly influence *T*_E_ values regarding MLR (adjusted *R* ^2^ = 0.822). For IR, a *P*‐value of 0.026 was calculated, whereas, for all other variables, the *P*‐value was below 0.001, pointing out a minor influence of irrigation on resulting *T*_E_ values than the variety or the growing temperatures (RN and TS). Compared to the reference of this MLR (AV, mm, no irrigation and RN 1), constantly hot temperatures during grain filling (temperature scenario hh) impact *T*_E_ values the most (regression coefficient = 3.401), followed by mh (3.062) and hm (2.525).

**Table 3:** Results of MLR and SMLR analysis with endset‐temperature (*T*_E_) being the dependent variable

MLR			SMLR		
Independent variable	Regression coefficient B	SE	Independent variable	Regression coefficient B	SE
Constant term	69.444	0.154	Constant term	72.110	0.118
hh***	3.401	0.154	mm	−3.029	0.126
mh***	3.062	0.154	SC***	1.858	0.101
hm***	2.525	0.154	MO:mh***	−1.605	0.161
RN***	1.407	0.109	IR:mh***	1.504	0.177
SC***	0.965	0.133	RN:mh***	1.414	0.199
MO**	−0.420	0.133	AV:hm***	1.328	0.156
IR*	0.244	0.109	MO:RN:IR***	1.315	0.169
(adjusted *R* ^2^ = 0.822)			RN:mh:IR***	−1.210	0.289
			MO:RN:mm***	1.189	0.206
			AV:hh***	1.159	0.169
			SC:mh***	−1.096	0.169
			RN***	1.024	0.102
			hm***	−0.551	0.151
			MO:hh**	0.479	0.171
			IR:hm**	−0.394	0.150
			RN:IR*	−0.276	0.135
			(adjusted *R* ^2^ = 0.930)		

*Note*: DSC temperature precision: ± 0.008 °C.

Abbreviations: AV, Avalon; SC, Scarlett; MO, Morex; RN, run; IR, irrigation; mm, moderate–moderate; mh, moderate–hot; hm, hot–moderate; hh, hot–hot; MLR, multiple linear regression; SMLR, stepwise multiple linear regression; DSC, differential scanning calorimetry.

* *P* < 0.05;

** *P* < 0.01;

*** *P* < 0.001.

Comparing MLR with SMLR (adjusted *R* ^2^ of 0.930) results (Table 3), the data show that the SC variety increases the *T*_E_ values (1.858) mainly independently of the temperature scenarios and the run (based on the calculated model with the const. term = 72.110). One exception is the temperature scenario hm, which, in combination with SC (SC:mh), significantly reduces the *T*_E_ values (−1.096). This contrasts with the MO variety, which delivered significantly lower *T*_E_ values than AV and SC in the MLR. The SMLR shows that MO is somewhat more dependent on the individual other factors: The combination MO:mh leads to significantly lower *T*_E_ values (−1.605) than the combinations MO:RN:IR (1.315), MO:RN:mm (1.185) or MO:hh (0.479), which are each associated with significantly higher *T*_E_ values. The (additional) dependence of the runs (RN) and thus the temperatures before GS 55 is particularly striking here. Regarding the AV variety, it can be seen that only the combinations hh and hm have an additional, varying influence on *T*_E_; thus, mm and mh are not significantly different (AV:hm 1.328; AV:hh 1.159).

Across varieties, it can be seen from the SMLR results that consistently moderate temperatures in the grain filling phase (temperature scenario mm) lead to significantly lower *T*_E_ values (mm: −3029). The same applies to the temperature scenario hm (−0.551), although the effect here is significant but much smaller than for mm. It is also shown that the impact of temperature before GS 55 (RN) is associated with significantly different *T*_E_ values across varieties (1.024). In particular, the combination of colder growing conditions until GS 55 (RN 2) with the temperature scenario mh is significantly associated with higher *T*_E_ values (RN:mh 1.414). The MO variety reacts significantly differently to the temperatures before GS 55 than the other varieties (MO:RN:mm; MO:RN:IR).

Concerning irrigation, the low influence on the *T*_E_ values identified in the MLR is confirmed in SMLR data. However, combining irrigation with the temperature scenario, mh is significantly associated with other *T*_E_ values across varieties (IR:mh 1.504). The *T*_E_ values significantly differ depending on the temperature before GS 55 (RN:mh:IR −1.210), which again emphasizes the importance of the temperature before GS 55.

### Impact of growth conditions on enthalpy (Δ*H* )

Table 4 shows the results of MLR and SMLR performed for the dependent variable Δ*H*. The underlying data are listed in the Supporting information (Table S3). Regarding MLR, the variables SC (*P* = 0.185), mh (*P* = 0.954) and hm (*P* = 0.242) do not significantly impact the tested Δ*H* values. It can be concluded from the relatively small adjusted *R* ^2^ (0.384) that the shown model can only describe the Δ*H* values to a minor extent. However, the MO variety has the greatest effect here and provides significantly lower Δ*H* values than the other two varieties. The variables RN, IR and hh also have a significant impact.

**Table 4:** Results of MLR and SMLR analysis with Δ*H* being the dependent variable

MLR			SMLR		
Independent variable	Regression coefficient B	SE	Independent variable	Regression coefficient B	SE
Constant term	3.217	0.037	Constant term	3.061	0.014
MO***	−0.297	0.032	MO:RN***	−0.353	0.032
hh*	−0.093	0.037	AV:RN***	0.157	0.031
RN**	−0.079	0.026	MO:IR***	−0.136	0.032
IR**	−0.073	0.026	AV:hh***	−0.132	0.041
(adjusted *R* ^2^ = 0.384)			(adjusted *R* ^2^ = 0.579)		

*Note*: DSC enthalpy precision: ± 0.08%.

Abbreviations: AV, Avalon; SC, Scarlett; MO, Morex; RN, run; IR, irrigation; mm, moderate–moderate; mh, moderate–hot; hm, hot–moderate; hh, hot–hot; MLR, multiple linear regression; SMLR, stepwise multiple linear regression; DSC, differential scanning calorimetry.

* *P* < 0.05;

** *P* < 0.01;

*** *P* < 0.001.

SMLR (adjusted *R* ^2^ = 0.579) results (Table 4) confirm the significant influences of the variables MO, RN, IR and hh identified in the MLR. However, the description of the resulting Δ*H* values is significantly better if the interactions with other variables are taken into account. Colder temperatures before GS 55 (RN 2) result in significantly lower values for MO than for AV (MO:RN −0.353; AV:RN 0.157). In relation to the constant term of this model (3.061), there are significantly lower Δ*H* values for the combination AV:hh. The same applies to the combination of irrigation and variety MO (MO:IR −0.136). With a comparatively low adjusted *R* ^2^, the dependencies of the Δ*H* values can only be worked out to a limited extent.

### Impact of growth conditions on B‐granule proportion

Table 5 shows the results of MLR and SMLR performed for the dependent variable B‐granule proportion. The underlying data are listed in the Supporting information (Table S4). Regarding MLR, all independent variables except RN significantly describe the B‐granule proportion values (adjusted *R* ^2^ = 0.669). Interpreting the values of corresponding regression coefficients, irrigation (IR; 4.559) is thus associated more than the other factors with the proportion of B‐granules. According to this calculation, the B‐granule proportion increases from an average of 5.025–9.584% if irrigation is conducted. Hot temperatures during the grain‐filling phase reduce the B‐granule proportion (compared to the mm scenario), as do the varieties Scarlett and Morex (compared to Avalon).

**Table 5:** Results of MLR and SMLR analysis with B‐granule proportion being the dependent variable

MLR			SMLR		
Independent variable	Regression coefficient B	SE	Independent variable	Regression coefficient B	SE
Constant term	5.025	0.376	Constant term	−0.171	0.133
IR***	4.559	0.284	IR	5.954	0.249
hh***	−3.196	0.402	AV:mm***	3.497	0.367
mh***	−2.985	0.402	RN:mh***	3.255	0.264
MO***	−2.542	0.348	RN:mm***	3.040	0.349
hm***	−2.419	0.402	IR:hm***	3.028	0.265
SC***	−1.607	0.348	AV:RN:hm***	−2.999	0.468
(adjusted *R* ^2^ = 0.669)			RN:IR***	−2.962	0.245
			AV:hm***	2.419	0.360
			MO:IR***	−2.390	0.273
			RN:hh***	2.335	0.260
			AV:RN:mm***	2.173	0.559
			IR:mm***	2.103	0.287
			MO:RN:mh***	1.984	0.452
			SC:mm***	1.439	0.306
			SC:IR***	−1.201	0.254
			MO:RN**	−0.759	0.284
			MO:hh*	0.592	0.287
			(adjusted *R* ^2^ = 0.930)		

*Note*: Mastersizer size determination accuracy: ± 0.6%.

Abbreviations: AV, Avalon; SC, Scarlett; MO, Morex; RN, run; IR, irrigation; mm, moderate–moderate; mh, moderate–hot; hm, hot–moderate; hh, hot–hot; MLR, multiple linear regression; SMLR, stepwise multiple linear regression; DSC, differential scanning calorimetry.

* *P* < 0.05;

** *P* < 0.01;

*** *P* < 0.001.

SMLR (adjusted *R* ^2^ = 0.930) results (Table 5) confirm that across varieties and temperature scenarios, irrigation (IR) has the greatest influence on the proportion of B‐granules (SMLR regression coefficient: 5.954). The cross‐variety significant effect of an increase in the proportion of B‐granules with irrigation is also significantly higher when irrigation occurs in combination with the temperature scenarios hm and mm (IR:hm 3.028; IR:mm 2.103). The negative coefficient of the combination of irrigation and temperature before GS 55 (RN:IR −2.962) shows that the B‐granule proportions of the irrigated starches are significantly lower in RN 2 than in RN 1. The MO and SC varieties also had significantly different B‐granule proportions under irrigation (MO:IR −2.390; SC:IR −1201).

The temperature before GS 55 also influenced the B‐granule proportion independently of irrigation: The combinations of RN and all temperature scenarios except hm yielded significantly different B‐granule values (RN:mh 3.255; RN:mm 3.040; RN:hh 2.335). The effect of irrigation and the temperature scenario during grain‐filling on the B‐granule proportion is thus significantly influenced by the temperature scenario before GS 55.

With regard to the varieties, the growing scenario led to significantly different B‐granule proportions for AV (AV:mm 3.497, AV:hm 2.419; AV:RN:hm −2.999; AV:RN:mm 2.173), irrespective of irrigation, but depending on RN. MO and SC, instead, yield different B‐granule proportion values if combined with irrigation (MO:IR −2.390; SC:IR −1.201).

## DISCUSSION

In the present study, we investigated the impact of heat and drought stress in a highly controlled environment on the B‐granule proportion and the gelatinization behavior of starch of three malting barley varieties. Because the plants were cultivated in pots in climate chambers, possible stress compensation strategies of the plant were inhibited, such as deeper root growth under drought stress, which is common in different crop varieties that can tolerate drought stress.^59^, ^60^ Simulating other forms of extreme weather, such as heavy rains or wind, was not possible in our experimental setup. Hence, all findings and conclusions are limited to the heat and drought scenarios simulated in the highly controlled climate chambers.

While irrigation *per se* did not significantly impact the gelatinization temperatures of the final starch, it did correlate with increased Δ*H* values (+0.074 J g^−1^ over all cultivars) and a higher ratio of B‐granules (+4.56% over all cultivars). For the starch structure, higher enthalpy values could indicate a higher percentage of crystalline layers or an altered crystal conformation in the starch granules.^23^, ^26^, ^29^ However, we cannot make a definitive statement because the present study did not analyze the molecular starch structure, such as the chain length distribution. To further understand the impact of climate conditions on the starch fine structure, future studies could include this analysis because this would allow a deeper understanding of the stress‐related regulation of the involved synthesis enzymes and the expected saccharification behavior of the final starch in the brewing process. Furthermore, the investigation of climate conditions on malting and brewing quality parameters such as percentage germination, enzymatic potential and extract yield could proof beneficial.

Heat stress was applied at different growth stages to answer whether heat stress from GS 55–75 has a higher impact on starch properties than heat stress from GS 75–93. Although continuous heat stress led to higher *T*_o_ and *T*_E_ values in all cultivars, no clear conclusion can be drawn as to whether early heat stress has more severe consequences than late heat stress. The highest impact was identified for constantly hot temperatures at 28 °C at daytime and 25 °C at night from GS 55–93 (temperature scenario hh). On average, *T*_o_ was increased by 3.12 °C and *T*_E_ was increased by 3.39 °C compared to the same cultivar at constantly moderate temperatures. Similar observations have been made for non‐malting barley and other starchy crops.^4^, ^44^ Even though all cultivars showed increased *T*_o_ and *T*_E_ values when exposed to heat stress conditions during grain‐filling, the severity of these effects differs between the three studied cultivars, highlighting the importance of considering the impact of different varieties. SC consistently delivered significantly higher *T*_E_ values (0.78 °C above average *T*_E_ of all cultivars), irrespective of temperature scenarios, whereas MO exhibited the lowest *T*_E_ values (0.61 °C below average of all cultivars) with significant dependencies on temperature and irrigation. In wheat, the application of heat stress within the first 8 days after anthesis had a greater effect than heat stress at a later point of the grain‐filling phase.^50^ The expression level of starch synthesis‐related enzymes is the highest between 6 and 12 days after anthesis,^32^ which is another indicator that the first half of the grain‐filling phase is more sensitive to heat stress. However, the same could not be observed in our experiments. Further research should investigate the most critical time for heat stress in malting barley concerning starch properties. This could involve analyzing the activity or expression levels of key starch synthesis enzymes to gain deeper insights into the underlying regulatory mechanisms that influence final starch structure.

Temperatures before GS 55 (RN) had a variety‐independent significant impact on gelatinization behavior, with colder temperatures (RN 1: 20.37 °C average day temperature; RN 2: 15.99 °C average day temperature) leading to an increase of *T*_o_ and *T*_E_ values by 1.80 and 1.41 °C, respectively. RN 2 also differs regarding the time needed until anthesis. This might be a result of the barley development being inhibited by low temperatures and temperature fluctuation.^61^ The temperature pre‐anthesis has less effect on ear development than heat stress post‐anthesis; this might be explained by the ear still being protected by the surrounding leaf,^62^ which could act as an isolation layer around the ear and actively cool it by transpiration. Furthermore, the expression of starch synthesis enzymes in the grain takes place after anthesis.^32^, ^63^ Nevertheless, it has previously been shown that adverse temperatures may lead to decreased grain numbers and grain weight in wheat and barley.^34^, ^35^, ^36^ The critical time for determining grain number and weight in barley begins several days before anthesis,^36^ suggesting that the critical time affecting starch synthesis and possibly the regulation of starch synthesis enzymes might also begin before anthesis. Understanding the importance of temperature from germination to the mature grain and its impact on the final starch structure could benefit future farming techniques, such as earlier sowing of spring barley varieties.

## Supporting information


**Data S1:** Onset values
**Data S2:** Endset values
**Data S3:** Enthalpy values
**Data S4:** B‐granule proportion values

## Data Availability

The data that supports the findings of this study are available in the supplementary material of this article.
