# Supplementary material for: The influence of systematic heat and drought applications at defined growth stages on malting barley starch properties
Source: J Sci Food Agric. 2025 Apr 2;105(8):4493–503. doi: 10.1002/jsfa.14183 (PMC12081993; doi:10.1002/jsfa.14183)
Supplement: Supplementary file 1 — Data S1: Onset values Data S2: Endset values Data S3: Enthalpy values Data S4: B‐granule proportion values [file JSFA-105-4493-s001.docx]

**Manuscript – JSFA-24-3225**

“The influence of systematic heat and drought applications at defined growth stages on malting barley starch properties”

Supporting Information

Table S1: Overview of Onset-(T_0_)-values in dependence of growing conditions (TS = temperature scenario; mm = moderat-moderat; mh = moderat-hot; hm = hot-moderate; hh = hot-hot; w =watered; d = drought; RN = Run) and barley variety (AV = Avalon; SC = Scarlett; MO = Morex); SD= standard deviation; n = 4

|  |  |  | **Onset-Temperature [°C]** | | | | | | | | |
| --- | --- | --- | --- | --- | --- | --- | --- | --- | --- | --- | --- |
|  |  |  | **AV** | |  | **SC** | |  | **MO** | |  |
| **TS** | **w/d** |  | **RN 1** | **RN 2** |  | **RN 1** | **RN 2** |  | **RN 1** | **RN 2** |  |
| mm | w |  | 59.02 | 60.31 |  | 59.95 | 60.86 |  | 58.51 | 62.01 |  |
|  |  | SD | 0.57 | 0.17 |  | 0.23 | 0.19 |  | 0.35 | 0.25 |  |
| mm | d |  | 59.33 | 59.58 |  | 59.72 | 61.78 |  | 58.53 | 60.80 |  |
|  |  | SD | 0.25 | 0.23 |  | 0.25 | 0.09 |  | 0.18 | 0.13 |  |
|  |  |  |  |  |  |  |  |  |  |  |  |
| mh | w |  | 61.43 | 60.98 |  | 61.73 | 61.62 |  | 59.75 | 61.99 |  |
|  |  | SD | 0.22 | 0.12 |  | 0.12 | 0.27 |  | 0.16 | 0.17 |  |
| mh | d |  | 60.87 | 61.23 |  | 60.97 | 62.73 |  | 59.58 | 61.27 |  |
|  |  | SD | 0.13 | 0.27 |  | 0.19 | 0.65 |  | 0.07 | 0.16 |  |
|  |  |  |  |  |  |  |  |  |  |  |  |
| hm | w |  | 59.44 | 62.63 |  | 59.97 | 61.60 |  | 57.66 | 62.17 |  |
|  |  | SD | 0.11 | 0.16 |  | 0.51 | 0.10 |  | 0.22 | 0.15 |  |
| hm | d |  | 60.47 | 62.41 |  | 61.61 | 62.77 |  | 58.43 | 62.26 |  |
|  |  | SD | 0.33 | 0.07 |  | 0.10 | 0.22 |  | 0.22 | 0.34 |  |
|  |  |  |  |  |  |  |  |  |  |  |  |
| hh | w |  | 62.95 | 63.83 |  | 61.61 | 64.53 |  | 60.47 | 64.32 |  |
|  |  | SD | 0.24 | 0.29 |  | 0.10 | 0.19 |  | 0.19 | 0.37 |  |
| hh | d |  | 62.59 | 63.66 |  | 63.48 | 64.69 |  | 59.67 | 62.66 |  |
|  |  | SD | 0.13 | 0.11 |  | 0.56 | 0.14 |  | 0.17 | 0.43 |  |

|  |  |  | **Endset-Temperature [°C]** | | | | | | | |  |
| --- | --- | --- | --- | --- | --- | --- | --- | --- | --- | --- | --- |
|  |  |  | **AV** | |  | **SC** | |  | **MO** | |  |
| **TS** | **w/d** |  | **RN 1** | **RN 2** |  | **RN 1** | **RN 2** |  | **RN 1** | **RN 2** |  |
| mm | w |  | 69.06 | 70.10 |  | 71.28 | 71.52 |  | 69.18 | 72.45 |  |
|  |  | SD | 0.58 | 0.19 |  | 0.20 | 0.34 |  | 0.45 | 0.22 |  |
| mm | d |  | 69.28 | 69.90 |  | 70.49 | 72.18 |  | 68.80 | 71.18 |  |
|  |  | SD | 0.49 | 0.31 |  | 0.51 | 0.20 |  | 0.14 | 0.24 |  |
|  |  |  |  |  |  |  |  |  |  |  |  |
| mh | w |  | 73.25 | 74.71 |  | 75.10 | 75.46 |  | 71.65 | 74.00 |  |
|  |  | SD | 0.29 | 0.26 |  | 0.45 | 0.29 |  | 0.39 | 0.28 |  |
| mh | d |  | 72.47 | 74.12 |  | 71.89 | 75.44 |  | 70.83 | 73.25 |  |
|  |  | SD | 0.10 | 0.56 |  | 0.60 | 0.42 |  | 0.22 | 0.42 |  |
|  |  |  |  |  |  |  |  |  |  |  |  |
| hm | w |  | 72.45 | 72.99 |  | 73.02 | 73.68 |  | 71.35 | 73.43 |  |
|  |  | SD | 0.24 | 0.22 |  | 0.41 | 0.18 |  | 0.43 | 0.25 |  |
| hm | d |  | 72.91 | 74.19 |  | 73.45 | 74.26 |  | 71.60 | 72.39 |  |
|  |  | SD | 0.21 | 0.31 |  | 0.16 | 0.32 |  | 0.41 | 0.36 |  |
|  |  |  |  |  |  |  |  |  |  |  |  |
| hh | w |  | 73.00 | 74.05 |  | 74.11 | 74.65 |  | 72.60 | 74.61 |  |
|  |  | SD | 0.18 | 0.18 |  | 0.44 | 0.35 |  | 0.39 | 0.32 |  |
| hh | d |  | 73.00 | 74.26 |  | 74.30 | 74.88 |  | 72.28 | 73.96 |  |
|  |  | SD | 0.18 | 0.20 |  | 0.64 | 0.21 |  | 0.29 | 0.59 |  |

Table S2: Overview of Endset-(T_E_)-values in dependence of growing conditions (TS = temperature scenario; mm = moderat-moderat; mh = moderat-hot; hm = hot-moderate; hh = hot-hot; w =watered; d = drought; RN = Run) and barley variety (AV = Avalon; SC = Scarlett; MO = Morex); SD= standard deviation; n = 4

|  |  |  | **Enthalpy [J/g]** | | | | | | | |  |
| --- | --- | --- | --- | --- | --- | --- | --- | --- | --- | --- | --- |
|  |  |  | **AV** | |  | **SC** | |  | **MO** | |  |
| **TS** | **w/d** |  | **RN 1** | **RN 2** |  | **RN 1** | **RN 2** |  | **RN 1** | **RN 2** |  |
| mm | w |  | 2.95 | 3.38 |  | 3.21 | 2.88 |  | 2.87 | 2.62 |  |
|  |  | SD | 0.08 | 0.05 |  | 0.39 | 0.05 |  | 0.12 | 0.09 |  |
| mm | d |  | 3.15 | 3.21 |  | 3.15 | 3.07 |  | 3.08 | 2.76 |  |
|  |  | SD | 0.11 | 0.06 |  | 0.04 | 0.11 |  | 0.08 | 0.12 |  |
|  |  |  |  |  |  |  |  |  |  |  |  |
| mh | w |  | 3.04 | 3.20 |  | 3.00 | 2.98 |  | 2.97 | 2.66 |  |
|  |  | SD | 0.16 | 0.13 |  | 0.02 | 0.12 |  | 0.13 | 0.09 |  |
| mh | d |  | 3.17 | 3.30 |  | 3.21 | 3.03 |  | 2.97 | 2.82 |  |
|  |  | SD | 0.09 | 0.09 |  | 0.11 | 0.10 |  | 0.07 | 0.11 |  |
|  |  |  |  |  |  |  |  |  |  |  |  |
| hm | w |  | 3.00 | 3.13 |  | 3.01 | 3.13 |  | 2.98 | 2.57 |  |
|  |  | SD | 0.15 | 0.14 |  | 0.22 | 0.09 |  | 0.17 | 0.08 |  |
| hm | d |  | 3.05 | 3.11 |  | 3.06 | 3.20 |  | 3.05 | 2.52 |  |
|  |  | SD | 0.05 | 0.15 |  | 0.13 | 0.11 |  | 0.02 | 0.15 |  |
|  |  |  |  |  |  |  |  |  |  |  |  |
| hh | w |  | 2.93 | 3.12 |  | 3.01 | 3.01 |  | 2.96 | 2.36 |  |
|  |  | SD | 0.04 | 0.09 |  | 0.12 | 0.04 |  | 0.09 | 0.11 |  |
| hh | d |  | 2.95 | 3.03 |  | 3.06 | 3.01 |  | 2.97 | 2.80 |  |
|  |  | SD | 0.05 | 0.04 |  | 0.14 | 0.12 |  | 0.03 | 0.15 |  |

Table S3: Overview of enthalpy-(ΔH)-values in dependence of growing conditions (TS = temperature scenario; mm = moderat-moderat; mh = moderat-hot; hm = hot-moderate; hh = hot-hot; w =watered; d = drought; RN = Run) and barley variety (AV = Avalon; SC = Scarlett; MO = Morex); SD= standard deviation; n = 4

|  |  |  | **B-granule proportion [%]** | | | | | | | |  |
| --- | --- | --- | --- | --- | --- | --- | --- | --- | --- | --- | --- |
|  |  |  | **AV** | |  | **SC** | |  | **MO** | |  |
| **TS** | **w/d** |  | **RN 1** | **RN 2** |  | **RN 1** | **RN 2** |  | **RN 1** | **RN 2** |  |
| mm | w |  | 11.50 | 13.94 |  | 8.35 | 8.38 |  | 5.13 | 4.37 |  |
|  |  | SD | 0.00 | 0.35 |  | 1.56 | 0.39 |  | 0.00 | 0.56 |  |
| mm | d |  | 3.21 | 8.24 |  | 1.26 | 3.93 |  | 0.00 | 2.77 |  |
|  |  | SD | 0.00 | 1.17 |  | 1.08 | 0.36 |  | 0.00 | 1.77 |  |
|  |  |  |  |  |  |  |  |  |  |  |  |
| mh | w |  | 5.90 | 5.64 |  | 4.17 | 4.59 |  | 2.80 | 3.54 |  |
|  |  | SD | 0.00 | 0.14 |  | 0.65 | 0.90 |  | 0.00 | 0.10 |  |
| mh | d |  | 0.00 | 3.70 |  | 0.01 | 3.22 |  | 0.00 | 1.69 |  |
|  |  | SD | 0.00 | 0.20 |  | 0.01 | 0.88 |  | 0.00 | 1.53 |  |
|  |  |  |  |  |  |  |  |  |  |  |  |
| hm | w |  | 12.50 | 4.52 |  | 7.20 | 4.13 |  | 6.17 | 5.36 |  |
|  |  | SD | 0.00 | 0.86 |  | 0.00 | 0.04 |  | 0.00 | 0.03 |  |
| hm | d |  | 0.98 | 0.00 |  | 0.00 | 0.21 |  | 0.59 | 0.39 |  |
|  |  | SD | 0.00 | 0.00 |  | 0.00 | 0.09 |  | 0.00 | 0.25 |  |
|  |  |  |  |  |  |  |  |  |  |  |  |
| hh | w |  | 4.56 | 5.77 |  | 5.51 | 4.28 |  | 4.60 | 2.36 |  |
|  |  | SD | 0.00 | 0.17 |  | 0.00 | 0.01 |  | 0.00 | 0.80 |  |
| hh | d |  | 0.00 | 3.05 |  | 0.00 | 1.56 |  | 0.00 | 2.05 |  |
|  |  | SD | 0.00 | 1.02 |  | 0.00 | 1.42 |  | 0.00 | 0.94 |  |

Table S4: Overview of B-granule-proportion-values in dependence of growing conditions (TS = temperature scenario; mm = moderat-moderat; mh = moderat-hot; hm = hot-moderate; hh = hot-hot; w =watered; d = drought; RN = Run) and barley variety (AV = Avalon; SC = Scarlett; MO = Morex); SD= standard deviation; n = 4
